# Bayesian Orthogonal Least Squares (BOLS) algorithm for reverse engineering of gene regulatory networks

**DOI:** 10.1186/1471-2105-8-251

**Published:** 2007-07-13

**Authors:** Chang Sik Kim

**Affiliations:** 1 Bioinformatics Group, Turku Centre for Computer Science, Turku, Finland

## Abstract

**Background:**

A reverse engineering of gene regulatory network with large number of genes and limited number of experimental data points is a computationally challenging task. In particular, reverse engineering using linear systems is an underdetermined and ill conditioned problem, i.e. the amount of microarray data is limited and the solution is very sensitive to noise in the data. Therefore, the reverse engineering of gene regulatory networks with large number of genes and limited number of data points requires rigorous optimization algorithm.

**Results:**

This study presents a novel algorithm for reverse engineering with linear systems. The proposed algorithm is a combination of the orthogonal least squares, second order derivative for network pruning, and Bayesian model comparison. In this study, the entire network is decomposed into a set of small networks that are defined as unit networks. The algorithm provides each unit network with P(D|H_i_), which is used as confidence level. The unit network with higher P(D|H_i_) has a higher confidence such that the unit network is correctly elucidated. Thus, the proposed algorithm is able to locate true positive interactions using P(D|H_i_), which is a unique property of the proposed algorithm.

The algorithm is evaluated with synthetic and *Saccharomyces cerevisiae *expression data using the dynamic Bayesian network. With synthetic data, it is shown that the performance of the algorithm depends on the number of genes, noise level, and the number of data points. With Yeast expression data, it is shown that there is remarkable number of known physical or genetic events among all interactions elucidated by the proposed algorithm.

The performance of the algorithm is compared with Sparse Bayesian Learning algorithm using both synthetic and *Saccharomyces cerevisiae *expression data sets. The comparison experiments show that the algorithm produces sparser solutions with less false positives than Sparse Bayesian Learning algorithm.

**Conclusion:**

From our evaluation experiments, we draw the conclusion as follows: 1) Simulation results show that the algorithm can be used to elucidate gene regulatory networks using limited number of experimental data points. 2) Simulation results also show that the algorithm is able to handle the problem with noisy data. 3) The experiment with Yeast expression data shows that the proposed algorithm reliably elucidates known physical or genetic events. 4) The comparison experiments show that the algorithm more efficiently performs than Sparse Bayesian Learning algorithm with noisy and limited number of data.

## Background

High-throughput technologies such as DNA microarrays provide the opportunity to elucidate the underlying complex cellular networks. There are now many genome-wide expression data sets available. As an initial step, several computational clustering analyses have been applied to expression data sets to find sets of co-expressed and potentially co-regulated genes [1-5]. As a next step, there have been efforts to elucidate gene regulatory networks (GRN) embedded in complex biological systems. A growing number of methods for reverse engineering of GRN have been reported as follows: Boolean networks [6,7], Bayesian networks [8-10], Algorithm for the Reconstruction of Accurate Cellular Networks (ARACNe) [11], linear models [12], neural networks [13], methods using ordinary differential equations [14,15], a sparse graphical Gaussian model [16], a method using a genetic algorithm [17], a method using Sparse Bayesian Learning (SBL) algorithm [18,19], and etc.

**Figure 1:**
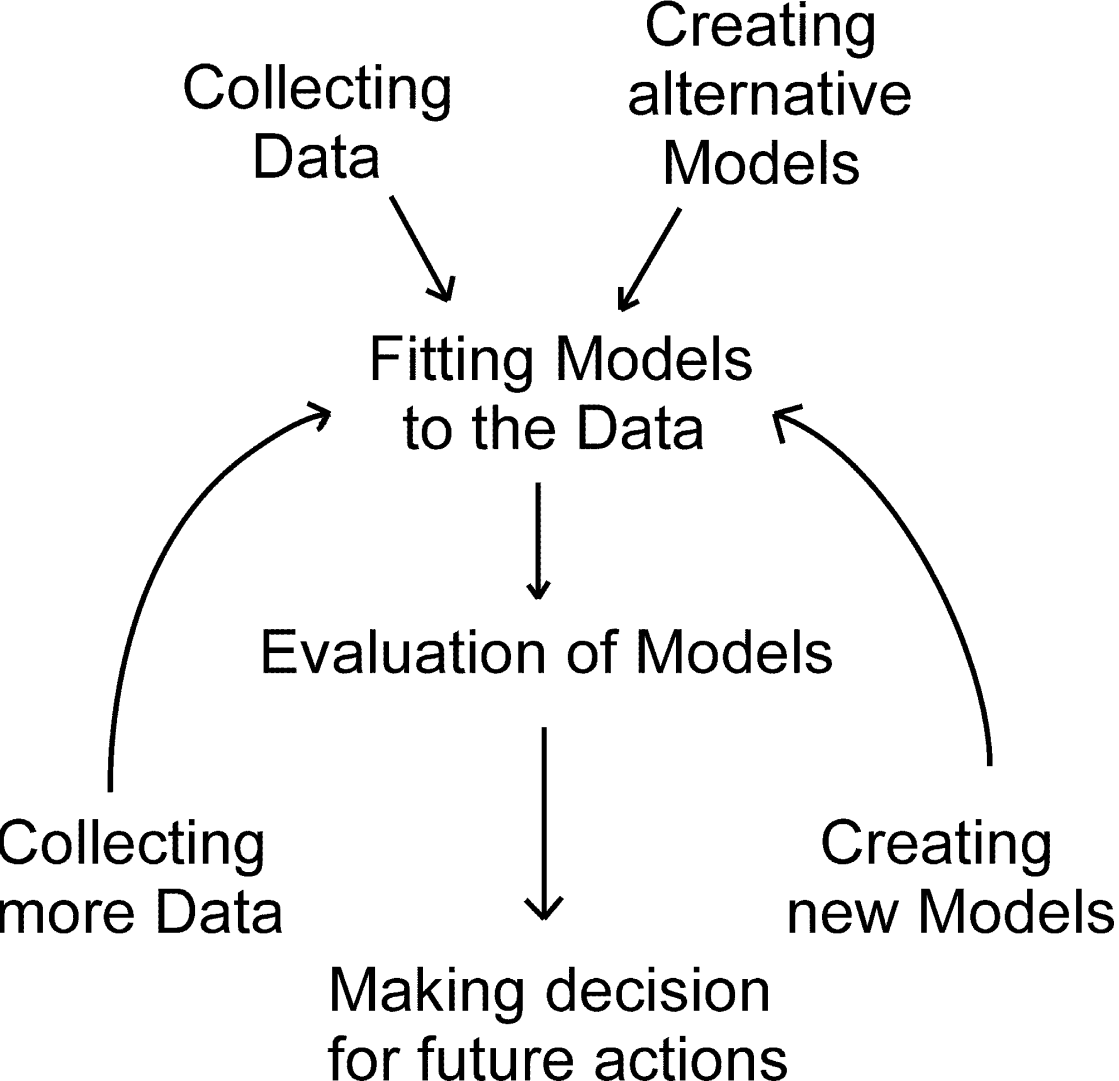
The Bayesian Orthogonal Least Squares algorithm could be used as a framework for gene regulatory study including the collecting and modeling of data.

In the reverse engineering of GRN, essential tasks are developing and comparing alternative GRN models to account for the data that are collected (Figure 1). There are two levels of inference involved in the task of data modeling process [20]. The first level of inference is fitting one of models to the data with an assumption that our chosen model is true. The second level of inference is the task of model comparison. It is desired to compare the alternative models with the help given by the data, and give some level of preference to the alternative models. Thus, the reverse engineering method should be used as a framework for fitting several different GRN models to the data to compare the models. For instance, there are several GRN modeling studies in which the reverse engineering algorithm could be applied: 1) system of ordinary differential equation (ODE) [15], 2) Dynamic Bayesian networks based methods (DBN) [10,18], 3) a linear stochastic differential equation for a transcriptional regulatory network [14], and etc. It is noted that these three models can be represented by linear systems. By a "linear system", we mean a system represented as a linear equation in matrix form such as Eq. 2 in methods section.

Microarrays have been used to measure genome-wide expression patterns during the cell cycle of different eukaryotic and prokaryotic cells. The review paper of Cooper and Shedden [21] presents various published microarray data sets, which have been interpreted as showing that a large number of genes are expressed in a cell-cycle-dependent manner. From this point of view, it is assumed that the underlying GRN is dependent upon cell-cyclic time. Therefore, this study uses the DBN because it can represent how the expression levels evolve over time.

In this paper, we address two main challenges in reverse engineering with linear systems and present a novel algorithm to overcome these difficulties. Firstly, reverse engineering of GRN will be computationally less challenging task if significantly large amount of experimental data is available. However, this is limited due to the expensive cost of microarray experiments. This problem makes the reverse engineering of GRN to be *underdetermined*, which means that there is substantially greater number of genes than the number of measurements. Secondly, reverse engineering of GRN with linear systems is *ill conditioned *because small relative changes in design matrix E in Eq. 2 due to the noise make substantially large changes in the solution. Therefore, the reverse engineering algorithm named as Bayesian orthogonal least squares (BOLS) is developed to overcome these difficulties. The BOLS method is created by combining three techniques: 1) Orthogonal Least Squares method (OLS) [22], 2) second order derivative for network pruning [23], and 3) Bayesian model comparison [20].

We evaluate the BOLS method by inferring GRN from both synthetic and Yeast expression data. We provide the performance comparison between BOLS and one of state-of-the-art reverse engineering methods, SBL algorithm [24]. The SBL algorithm has been recently used in GRN studies with linear systems [18,19]. For evaluation with Yeast expression data, we validate the inferred GRN using the information from the database that contains large data sets of known biological interactions.

## Results and discussion

### Case study 1: *In silico *experiment

**Figure 2:**
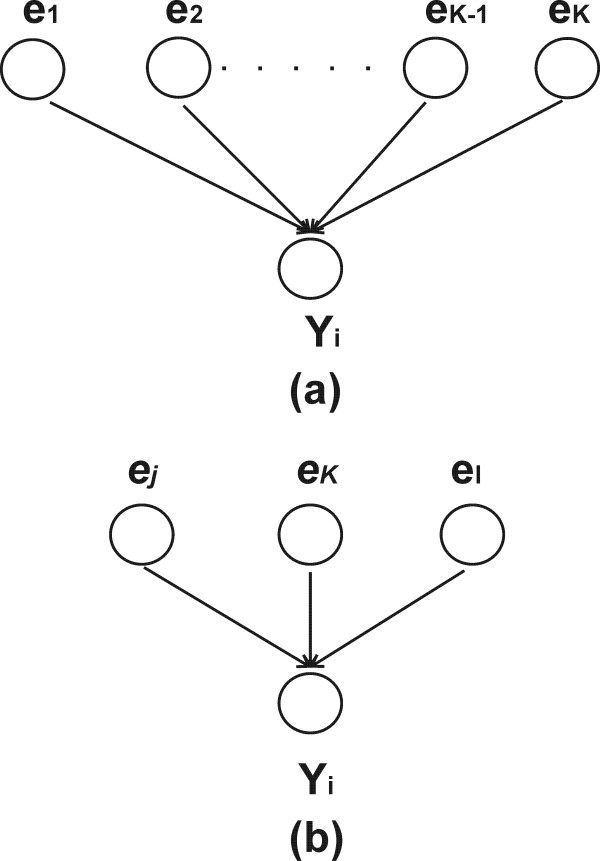
The schematic of unit network. (a) Input unit network consisting of target gene **Y**_**i **_and all other genes as regulator candidates. (b) Output unit network consisting of target gene **Y**_**i **_and its most probable regulators.

Our *in silico *experiment follows the methodology for the generation of synthetic expression dataset for systems of DBN as used in Rogers and Girolami [19]. We generate synthetic networks using power-law distribution. To create a network structure, we decompose the entire network into a set of small networks that are defined as unit network and proceed a unit network by a unit network. Figure 2 presents a unit network consisting of a target gene and a list of genes as regulators. It should be noted that there is no requirement for the network to be acyclic. All created unit networks will be combined to create a whole GRN. The combination of all (or selected) unit networks is straightforward process based on the definition of a graph (see Methods section). This unit network approach is similar to the approach adopted in Bayesian network based methods [9,25] and SBL based method [19]. For each target gene, we sample the number of genes (m_i_) regulating this target gene from the approximate power distribution

P(mi)={M−1mi−ηmmin⁡≤mi≤mmax⁡0otherwise
 MathType@MTEF@5@5@+=feaafiart1ev1aaatCvAUfKttLearuWrP9MDH5MBPbIqV92AaeXatLxBI9gBaebbnrfifHhDYfgasaacH8akY=wiFfYdH8Gipec8Eeeu0xXdbba9frFj0=OqFfea0dXdd9vqai=hGuQ8kuc9pgc9s8qqaq=dirpe0xb9q8qiLsFr0=vr0=vr0dc8meaabaqaciaacaGaaeqabaqabeGadaaakeaacqWGqbaudaqadaqaaiabd2gaTnaaBaaaleaacqWGPbqAaeqaaaGccaGLOaGaayzkaaGaeyypa0ZaaiqaaeaafaqaaeGacaaabaGaemyta00aaWbaaSqabeaacqGHsislcqaIXaqmaaGccqWGTbqBdaqhaaWcbaGaemyAaKgabaGaeyOeI0ccciGae83TdGgaaaGcbaGaemyBa02aaSbaaSqaaiGbc2gaTjabcMgaPjabc6gaUbqabaGccqGHKjYOcqWGTbqBdaWgaaWcbaGaemyAaKgabeaakiabgsMiJkabd2gaTnaaBaaaleaacyGGTbqBcqGGHbqycqGG4baEaeqaaaGcbaGaeGimaadabaGaem4Ba8MaemiDaqNaemiAaGMaemyzauMaemOCaiNaem4DaCNaemyAaKMaem4CamNaemyzaugaaaGaay5Eaaaaaa@5C98@

where the normalization constant is given by

M=∑i=mmin⁡mmax⁡mi−η.
 MathType@MTEF@5@5@+=feaafiart1ev1aaatCvAUfKttLearuWrP9MDH5MBPbIqV92AaeXatLxBI9gBaebbnrfifHhDYfgasaacH8akY=wiFfYdH8Gipec8Eeeu0xXdbba9frFj0=OqFfea0dXdd9vqai=hGuQ8kuc9pgc9s8qqaq=dirpe0xb9q8qiLsFr0=vr0=vr0dc8meaabaqaciaacaGaaeqabaqabeGadaaakeaacqWGnbqtcqGH9aqpdaaeWbqaaiabd2gaTnaaDaaaleaacqWGPbqAaeaacqGHsisliiGacqWF3oaAaaaabaGaemyAaKMaeyypa0JaemyBa02aaSbaaWqaaiGbc2gaTjabcMgaPjabc6gaUbqabaaaleaacqWGTbqBdaWgaaadbaGagiyBa0MaeiyyaeMaeiiEaGhabeaaa0GaeyyeIuoakiabc6caUaaa@4559@

Following Rogers and Girolami [19], Wagner [26], and Rice et al. [27], the constant *η *is set to 2.5. m_max _(or m_min_) is the maximum (or minimum) number of allowed regulator genes in a unit network respectively. The condition m_max _<< K (the number of genes) ensures the sparseness of the synthetic network. Note that m_max _is set to 4 for all experiments. The more details of creating synthetic network can be found from the supplementary information of Rogers and Girolami's study [19]. In this study, the Matlab code from Rogers and Girolami's study for creating synthetic network is used, which is available from [28]. We first randomly generate the synthetic networks unit network by unit network to combine them for a whole GRN, and then we generate the synthetic expression data with randomly generated synthetic GRN. Using these synthetic expression data, we infer the network with BOLS and the simulated data set. It should be noted that BOLS and the generation method of synthetic networks are not cooperative to work because the generation and inference of networks are completely separate processes.

In this experiment, the synthetic expression data is generated based on DBN using Eq. 1, in which the expression data are evolved over the time. However, as the simulation process is continued over the time, the expression data diverge by constantly either increasing or decreasing. Thus, we collect a single time point only after the expression data are simulated for a certain period of time (from t = 0 to t = T) to avoid expression levels being too high. We proceed in a single time point by a single time point manner. For a single time point, the generation of expression data is started with initial synthetic data. For each gene, the initial condition is assigned with random number between 0 and 1. With given initial condition, we simulate the expression data for each gene i from t = 0 to t = T using Eq. 1. We take the measure with t = T-1 for design matrix E and with t = T for Y_i _in Eq. 2. We repeat these process N times to collect N data points and apply the reverse engineering algorithms to reconstruct the synthetic network.

We make the performance comparison between BOLS and SBL methods to show the efficiency of BOLS method. The SBL method is one of the state-of-the-art algorithms, which has been recently applied to GRN studies with linear systems [18,19]. We use the Matlab code of SBL algorithm that is available from [29]. At first, we investigate the effect of the number of data points (N = 20, 40, 60, 80, 100) and noise (*ε *= 0.01, 0.05, 0.1) on the performance using synthetic GRN and expression data with fixed number of genes (K = 100). Sensitivity and complementary specificity are used as measures for the performance of the algorithm [9,19,27]. We compute the sensitivity = TP/(TP + FN) and the complementary specificity = FP/(TN + FP), where TP is the number of true positive, FN is the number of false negative, FP is the number of false positive, and TN is the number of true negative. In other words, the sensitivity is the proportion of recovered true positive interactions and the complementary specificity is the proportion of false positive interactions. To investigate the variability of test results, we have run both algorithms 20 times with same control parameters, i.e. the number of data points, noise, and etc. For each run, new random synthetic network has been created. We find that the sensitivity and the complementary specificity are constant over 20 experiments with small variability (see Table 1). The systematic effect of noise and the number of data points on the performance can be analyzed from Table 1. As the number of data points increases with the fixed number of genes and noise level *ε*, the performance of both algorithms increase. As noise level *ε *increases, the performance of both algorithms decreases. For the number of data points ≥ 80, the sensitivity of SBL is slightly greater than BOLS and the complementary specificity of SBL is significantly greater than the ones of BOLS. It means that BOLS algorithm produces significantly smaller proportions of false positive interactions than SBL algorithm. It should be noted that results from SBL algorithm with N = 20, 40, and 60 are not available because the Matlab code of SBL algorithm dose not run when the number of data points N is relatively low. SBL algorithm includes the Cholesky factorization of their Hessian matrix that is required to be positive definite. Note that the Hessian matrix is consisted of design matrix and hyperparameters [24]. When the number of data points is relatively smaller than the number of genes in the data set, this Hessian matrix becomes non positive definite. Our experiments show that SBL algorithm is not suitable for the reverse engineering with limited number of data points and it doesn't even run with significantly limited number of data points. On the other hand, BOLS algorithm produces relatively small proportion of FP interactions with limited number of data points. It should be noted that Rogers and Girolami [19] generate 2R expression levels for each gene in each knock-out experiment-R in the normal (wild type) system and R in the knock-out (mutant) system. In a network of K genes, in which each is knocked out individually, they have 2RK data points. Since the evaluation of BOLS with Rogers and Girolami's knockout approach [19] is beyond the scope of the objectives of our study, i.e. the *underdetermined *problem using DBN model, we provide the comparisons between BOLS and SBL based on their approach as Additional file 1.

**Table 1:** Sensitivity and complementary specificity for BOLS and SBL algorithms

*ε*	Sensitivity		C. Specificity	
	BOLS	SBL	BOLS	SBL
**N = 20**				
				
0.01	0.9676 ± 0.0192	_	0.0009 ± 6.171e-4	_
0.05	0.9328 ± 0.0215	_	0.0015 ± 6.143e-4	_
0.10	0.8900 ± 0.0255	_	0.0022 ± 5.095e-4	_
				
**N = 40**				
				
0.01	0.9879 ± 0.0089	_	0.0002 ± 1.731e-4	_
0.05	0.9632 ± 0.0074	_	0.0005 ± 2.146e-4	_
0.10	0.9371 ± 0.0235	_	0.0010 ± 3.919e-4	_
				
**N = 60**				
				
0.01	0.9863 ± 0.0082	_	0.0001 ± 1.053e-4	_
0.05	0.9720 ± 0.0097	_	0.0004 ± 2.066e-4	_
0.10	0.9447 ± 0.0127	_	0.0008 ± 2.326e-4	_
				
**N = 80**				
				
0.01	0.9872 ± 0.0095	0.9976 ± 0.0039	0.0002 ± 1.693e-4	0.3007 ± 0.0082
0.05	0.9694 ± 0.0110	0.9896 ± 0.0064	0.0005 ± 2.054e-4	0.3147 ± 0.0072
0.10	0.9448 ± 0.0201	0.9814 ± 0.0113	0.0008 ± 3.727e-4	0.3270 ± 0.0084
				
**N = 100**				
				
0.01	0.9883 ± 0.0084	0.9988 ± 0.0027	0.0002 ± 1.218e-4	0.2953 ± 0.0063
0.05	0.9694 ± 0.0121	0.9915 ± 0.0075	0.0004 ± 1.883e-4	0.3030 ± 0.0099
0.10	0.9517 ± 0.0183	0.9843 ± 0.0089	0.0006 ± 2.494e-4	0.3095 ± 0.0075

N is the number of data points, and *ε *the noise level. m_max _is set to 4 and the number of genes K 100.

**Figure 3:**
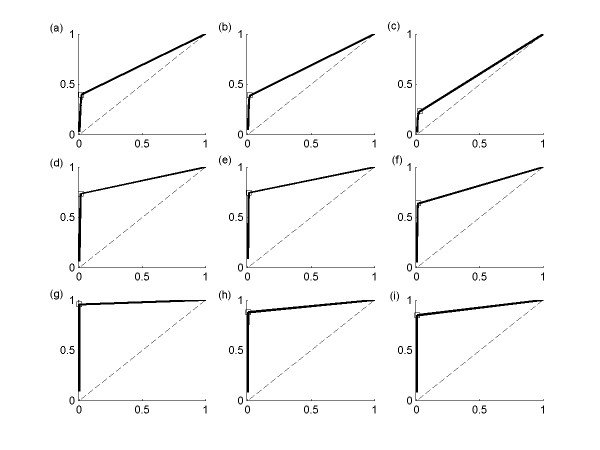
ROC analysis of BOLS output with K = 100. (a) N = 5 and *ε *= 0.01, (b) N = 5 and *ε *= 0.05, (c) N = 5 and *ε *= 0.1, (d) N = 10 and *ε *= 0.01, (e) N = 10 and *ε *= 0.05, (f) N = 10 and *ε *= 0.1, (g) N = 15 and *ε *= 0.01, (h) N = 15 and *ε *= 0.05, (i) N = 15 and *ε *= 0.1. For all Figures, the x-axis corresponds to the complementary specificity, the y-axis sensitivity.

**Figure 4:**
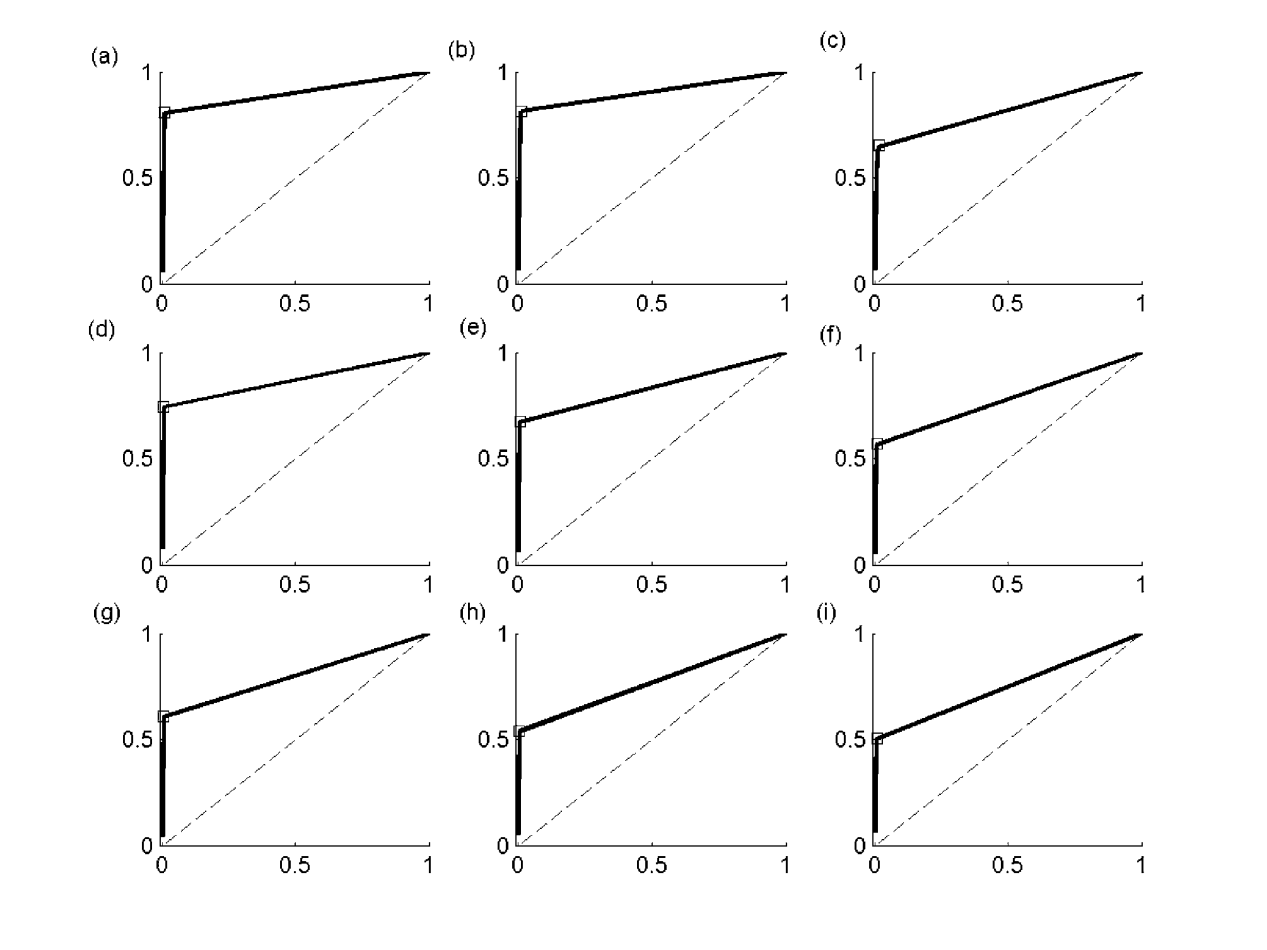
ROC analysis of BOLS output with N = 10. (a) K = 100 and *ε *= 0.01, (b) K = 100 and *ε *= 0.05, (c) K = 100 and *ε *= 0.1, (d) K = 200 and *ε *= 0.01, (e) K = 200 and *ε *= 0.05, (f) K = 200 and *ε *= 0.1, (g) K = 300 and *ε *= 0.01, (h) K = 300 and *ε *= 0.05, (i) K = 300 and *ε *= 0.1. For all Figures, the x-axis corresponds to the complementary specificity, the y-axis sensitivity.

We use the receiver operating characteristic (ROC) analysis [19] to characterize the trade-off between the proportions of true and false positive interactions with limited number of data points (N < 20 and K = 100) in Figure 3. This ROC analysis shows that BOLS algorithm produces the solution with extremely small proportion of false positive interactions when the number of data points is extremely small. We also analyze the effect of the number of gene K with limited number of data points N = 10. In Figure 4, it is shown that the performance of BOLS algorithm decreases as K increases from 100 to 300. Therefore, it can be concluded that performance of the proposed algorithm is dependent on K, N, and *ε*.

**Figure 5:**
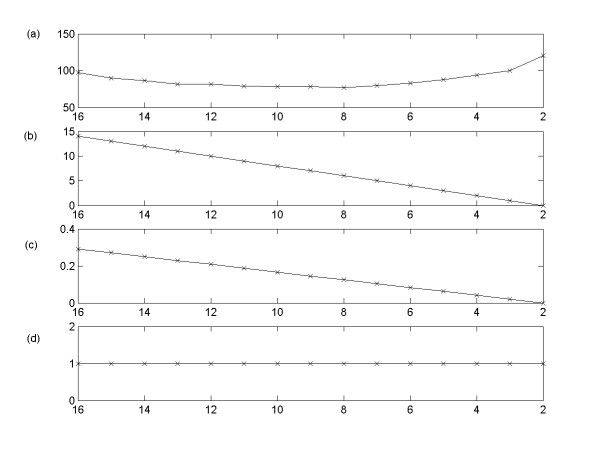
The changes of performance of BOLS as the network pruning step proceeds. The simulation experiment is done with N = 50, K = 20, and *ε *= 0.1. In these Figures, we concentrate on an output unit-network that has the highest logP(D|H_i_) among all output unit networks. For all Figures, the x-axis corresponds to the number of inferred interactions: as the network pruning proceeds, the number of inferred interactions in unit network decreases. Each y-axis corresponds to (a) logP(D|H_i_), (b) the number of errors (FP+FN), (c) the complementary specificity, (d) the sensitivity.

From the experiments, it is shown that BOLS produce solutions with significantly low complementary specificity regardless of K, N, and *ε*, because Bayesian model selection scheme is efficiently enough to discover the optimal solution. Since we do not have any information on noise in the data, we completely over-fit the data to DBN model using OLS as a first step. Then, we remove the unnecessary inferred parameters (the inferred parameters that are related with "noise") to obtain the optimal solution by a trade-off between minimizing the natural complexity of inferred GRN and minimizing the data misfit. As the complexity of inferred GRN decreases with network pruning process, we use the Bayesian model selection to select the most optimal solution. It should be noted that the Bayesian model selection includes *Occam's factor*, which automatically suppresses the tendency to discover spurious structure in data. Thus, we can say that BOLS is efficient to infer GRN with significant small portion of FP interactions with the noisy and limited number of data set for DBN. In Figure 5, we present an example showing that the performance of BOLS increases as the network pruning step proceeds. We first generate the synthetic networks of 50 genes (N) and then simulate 20 data points (K) with this networks and noise level *ε *= 0.1. We concentrate on an output unit-network with highest evidence value logP(D|H_i_) for evaluation. It should be reminded that as the network pruning continues the number of inferred interactions in the unit network decreases. Figure 5b shows that the number of errors (FP+FN) decreases, as the network pruning proceeds. The complementary specificity also decreases along the network pruning (Figure 5c). On the other hand, the sensitivity remains constant, which is equal to 1 (Figure 5d). It means that the over-fitted solutions after OLS step contain only TP and FP interactions (no FN interactions). It is observed that the number of errors and the complementary specificity converge at 0 as the network pruning process proceeds, in which the unit network has the highest evidence logP(D|H_i_) (Figure 5a). Therefore, it can be concluded that the OLS method can cope with underdetermined problems using noisy data, provided that the method is combined with network pruning process and Bayesian model selection techniques.

**Figure 6:**
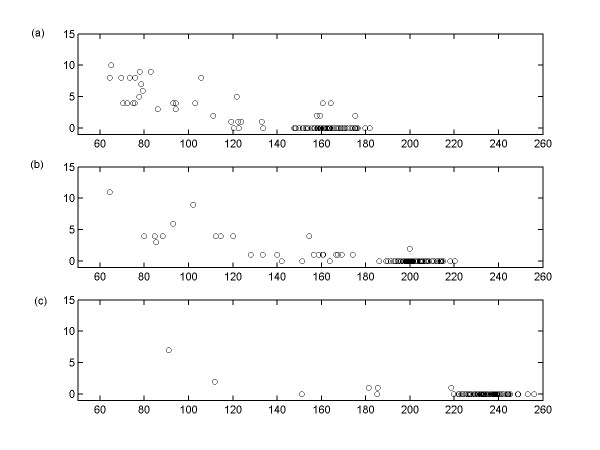
The relationship between the evidence value P(D|H_i_) and the number of errors for unit networks, where K = 100, *ε *= 1.0e-2, and m_max _= 4. (a) N = 10, (b) N = 15, (c) N = 20. For all Figures, the x-axis corresponds to log(P(D|H_i_)), the y-axis the number of errors

The BOLS algorithm should be run K times producing K unit networks, which are combined to build the whole network. Each unit network is assigned with P(D|H_i_). For each unit network, we compute the number of errors = FN + FP. The relationship between P(D|H_i_) and the number of errors for each unit-networks is shown in Figure 6. The number of errors decreases as the number of data points increases on the synthetic data. Unit networks with higher P(D|H_i_)s are more accurate than those with lower P(D|H_i_)s. This signifies that unit networks with higher P(D|H_i_)s have a higher confidence that unit networks are correctly reconstructed. Thus, when low numbers of data points and extremely high numbers of genes are given, the algorithm should be able to recover a partially correct network with unit networks only having relatively high P(D|H_i_)s. It should be noted that BOLS algorithm does not provide the confidence levels among interactions inside unit network. However, it can be noticed from Figure 6 that many unit networks with relatively high evidence values have zeroed number of incorrectly inferred interactions. Therefore, the evidence values for unit networks are efficient enough to cope with problems for locating unit networks without FP or FN interactions.

### Case study 2: Application of BOLS to *Saccharomyces cerevisiae *data

To evaluate our algorithm for reverse engineering of GRN, we use the microarray data from Spellman *et al*. [30], in which they created three different data sets using three different synchronization techniques. We focus our analysis using the data set produced by *α *factor arrest method. Here we concentrate on our study with the expression data set of 20 genes known to be involved in cell cycle regulation [31]. Thus, we have a data set with 20 genes and 17 data points in this experiment. Based on the previous simulation experiment, it is expected to have sensitivity as 0.967 (*ε *= 0.01), 0.937 (*ε *= 0.05), 0.919 (*ε *= 0.1) and complementary specificity as 0.022 (*ε *= 0.01), 0.019 (*ε *= 0.05), 0.029 (*ε *= 0.1), respectively. It means that the output is expected to have approximately less than 3% of complementary specificity if we assume that *ε *≤ 0.1, the sparseness m_max _of GRN = 4, and etc.

The output unit networks using this data set are presented in Table 2 including the P(D|H_i_) of unit networks, in which there are 107 inferred interactions from BOLS. Among those interactions, 45 of them are identified as physical or genetic interactions from the BioGRID database [32]. Later in this section, we provide the logical basis such that some of unidentified interactions might be possible physical or genetic events. BioGRID is a freely accessible database including physical or genetic interactions from the *Saccharomyces cerevisiae *available at [33]. The output in Table 2 shows that the interactions with higher P(D|H_i_)s have higher likelihood of having known physical or genetic interactions identified from the BioGRID than the interactions with lower P(D|H_i_)s: 1) Among the elucidated interactions with P(D|H_i_) > 66^th ^percentile of all P(D|H_i_)s, 23 of them are identified as physical or genetic interactions from BioGRID database. 2) Among the elucidated interactions with 66^th ^percentile > P(D|H_i_)s > 33^rd ^percentile, 14 of them are identified as physical or genetic interactions, 3) Among the elucidated interactions with P(D|H_i_)s < 33^rd ^percentile, 8 of them are identified as physical or genetic interactions. This could be explained from the previous simulation experiment showing that BOLS has less false positive interactions with relatively high P(D|H_i_) than with low P(D|H_i_). It is noted that the overall values of P(D|H_i_) in Table 2 are relative small compared to the ones in Figure 6. It should be noted that number of genes is set to 20 in Table 2 and the number of genes is set to 100 in Figure 6. From Figure 6, it is also noted that the overall values of P(D|H_i_) decreases as the number of data points decreases. It is also found that the overall values of P(D|H_i_) decreases as the noise level increases. Therefore, the overall values of P(D|H_i_) can be relatively different depending on the number of genes, noise level, and the number of data points.

**Table 2:** The output unit networks of BOLS with log(P(D|H_i_))

Target	Regulators			log(P(D|H_i_))
Cln3	Cln2 (X)	Clb2 (X)	Cln1 (X)	51.10881
Cdc20	Clb1 (_)	Cln1 (_)		39.92276
Swi5	Clb1 (_)	Clb6 (_)		38.47693
Clb1	Clb2 (X)	Swi6 (_)		38.11346
Clb2	Clb1 (X)	Clb6 (_)		37.67802
Cln2	Clb6 (X)	Sic1 (X)	Clb1 (_)	37.24355
Clb5	Clb6 (X)	Sic1 (X)	Clb2 (X)	34.73177
	Cdc20 (X)	Swi4 (X)	Cln3 (X)	
	Hct1 (X)	Swi6 (X)	Cln2 (_)	
	Clb1 (_)	Mcm1 (_)	Mbp1 (_)	
	Swi5 (_)	Clb4 (_)		
Cln1	Swi4 (X)	Sic1 (X)		34.6732
Cdc28	Hct1 (X)	Clb2 (X)	Sic1 (X)	34.09275
	Swi5 (X)	Cln3 (X)	Swi6 (X)	
	Cln1 (X)	Cdc20 (X)	Clb1 (X)	
	Cln2 (X)	Clb4 (X)	Cdc53 (_)	
	Skp1 (_)	Mbp1 (_)	Cdc34 (_)	
	Clb6 (_)	Mcm1 (_)		
Clb6	Cln3 (X)	Swi4 (_)		33.44521
Swi6	Cln3 (X)	Mbp1 (X)	Sic1 (X)	31.96447
	Cln2 (X)	Swi4 (X)	Clb2 (X)	
	Clb5 (X)	Hct1 (_)	Mcm1 (_)	
	Cdc34 (_)	Clb4 (_)	Cdc20 (_)	
	Swi5 (_)	Skp1 (_)		
Swi4	Cln3 (X)	Clb6 (_)		31.85847
Mcm1	Cdc28 (_)	Cln3 (_)	Skp1 (_)	27.06636
	Swi6 (_)	Cdc53 (_)	Clb2 (_)	
	Cdc20 (_)	Mbp1 (_)	Clb1 (_)	
	Clb6 (_)	Clb5 (_)	Cln1 (_)	
	Cln2 (_)	Clb4 (_)		
Sic1	Cdc20 (X)	Swi4 (_)		26.16702
Hct1	Swi4 (X)	Cln3 (_)		25.80372
Mbp1	Clb1 (_)	Mcm1 (_)		25.54062
Cdc53	Cln2 (X)	Sic1 (X)		24.09588
Skp1	Cdc53 (X)	Cdc34 (X)	Swi6 (_)	24.0043
	Clb6 (_)	Sic1 (_)	Mbp1 (_)	
	Cln3 (_)	Swi5 (_)	Cdc28 (_)	
	Clb5 (_)	Clb4 (_)	Cln1 (_)	
	Cdc20 (_)	Clb1 (_)	Hct1 (_)	
	Mcm1 (_)			
Clb4	Mbp1 (_)	Hct1 (_)		23.45985
Cdc34	Cln1 (X)	Sic1 (X)		23.20024

(X)s indicate that the regulator gene is identified as having known interactions with its target gene from the BioGRID database, which is a freely accessible database of physical and genetic interactions available at [33].

We pool both physical and genetic interactions from BioGRID to validate the output interactions in this experiment. The rationale for this pooling can be described as follows. Several proteins join together to form multi-protein complex having certain functions or regulating other proteins. For example, SCF complex consists of Skp, Cullin, and F-box proteins, which promotes G1-S transition by targeting G1 cyclins and Cln-Cdk inhibitor Sic1 for degradation. From Breeden's [34] review study, it is known that cell cycle regulated complexes with at least one sub-unit are regulated at the transcript level. This means that certain protein complexes might regulate other complexes with time delay. With only expression data sets, we only have information that which proteins are present for certain time. In terms of efficiency and logical order, it is assumed that the cell only makes the proteins when it is needed. If the proteins are made all the time, the cell could be inefficient in an environment without the substrates of the protein [21]. From these points of view, there can be two possible cases to be considered when certain two proteins are present: 1) two proteins form a multi-protein complex by interacting each other, 2) One protein might form a complex with some other proteins. This complex might regulates the other protein that could also form a protein complex. Therefore, those two types of interactions can be pooled together to validate the output interactions.

Unit-networks having relatively many identified physical or genetic interactions are the ones with Cln3, Cln2, Clb5, Cln1, Cdc28, Swi6, Cdc53 and Cdc34 as target genes. For example, we present a unit network with Cdc28 as a target gene. Cdc28 is identified as having physical or genetic interactions with Cln1 [35-40], Cln2 [36-49], Cln3 [47,50-55], Clb1 [35,56,57], Clb2 [35,38,41,58-64], Clb4 [56,57,61], Hct1 [38,53,63,65-67], Sic1 [37,53,61,68-71], Cdc20 [53,67], Swi5 [53], and Swi6 [53,72]. Cdc28 is a catalytic subunit of the main cell cycle cyclin-dependent kinase (CDK), which alternatively associates with G1 cyclins (Cln1, Cln2, and Cln3) and G2/M cyclins (Clb1, Clb2, and Clb4) that direct the CDK to specific substrates. Hct1 (Cdh1) and Cdc20 are cell cycle regulated activators of the anaphase-promoting complex/cyclosome (APC/C), which direct ubiquitination of mitotic cyclins. One of Cdc28's Gene Ontology definitions gives another evidence that Cdc28 might have associations with Hct1 and Cdc20, because Cdc28 is involved in the progression from G1 phase to S phase of the mitotic cell cycle. Sic1 is an inhibitor of Cdc28-Clb kinase complexes that controls G1/S phase transition, which prevents premature S phase and ensuring genomic integrity.

Among the list of genes inside Cdc28 unit network, there are several genes not identified as having physical or genetic interactions with Cdc28 from the BioGRID: Clb6, Mbp1, Mcm1, Cdc34, Cdc53 and Skp1. However, the definitions of these genes from the BioGRID give enough evidences such that some of them indirectly interact with Cdc28. For example, Clb6 is a B-type cyclin involved in DNA replication during S phase, which activates Cdc28 to promote initiation of DNA synthesis. Clb6 also has a role for the formation of mitotic spindles along with Clb3 and Clb4. Thus, Clb6 indirectly regulates Cdc28 along with Clb4. Cdc53, Cdc34 and Skp1 also indirectly regulate Cdc28 through Sic1. They form a structural protein of SCF complexes, called cullin, with an F-box protein. The SCF promotes the G1-S transition by targeting G1 cyclins and the Cln-Cdk inhibitor Sic1 for degradation. Mbp1 is a transcription factor involved in regulation of cell cycle progression from G1 to S phase, which forms a complex with Swi6 that binds to Mull cell cycle box regulatory element in promoters of DNA synthesis genes. Thus, Mbp1 is associated with Cdc28 by forming a complex with Swi6.

For another example, we present a unit network having Clb5 as target gene. Clb5 is identified as having physical or genetic interactions with Clb6 [73-79], Sic1 [41,61,71,80-82], Clb2 [83], Cdc20 [84], Cln3 [75], Hct1 [63,85], Swi4 [75,86], and Swi6 [86].

Among the list of genes inside Clb5 unit network, there are several genes not identified as having physical or genetic interactions with Clb5: Cln2, Clb1, Mcm1, Mbp1, Swi5 and Clb4. However, there are evidences that some of genes in the list might have indirect interactions with Clb5. For example, Clb4 has an association with Clb5, because Clb5 is a B-type cyclin involved in DNA replication during S phase and has a role for the formation of mitotic spindles along with Clb3 and Clb4. For another example, Mbp1 and Clb5 have an indirect interaction. Mbp1 is a transcription factor involved in regulation of cell cycle progression from G1 to S phase, which forms a complex with Swi6 that binds to MluI cell cycle box regulatory element in promoters of DNA synthesis genes. It is already identified that Swi6 regulates Clb5. Thus, Mbp1 indirectly regulate Clb5 through Swi6.

**Figure 7:**
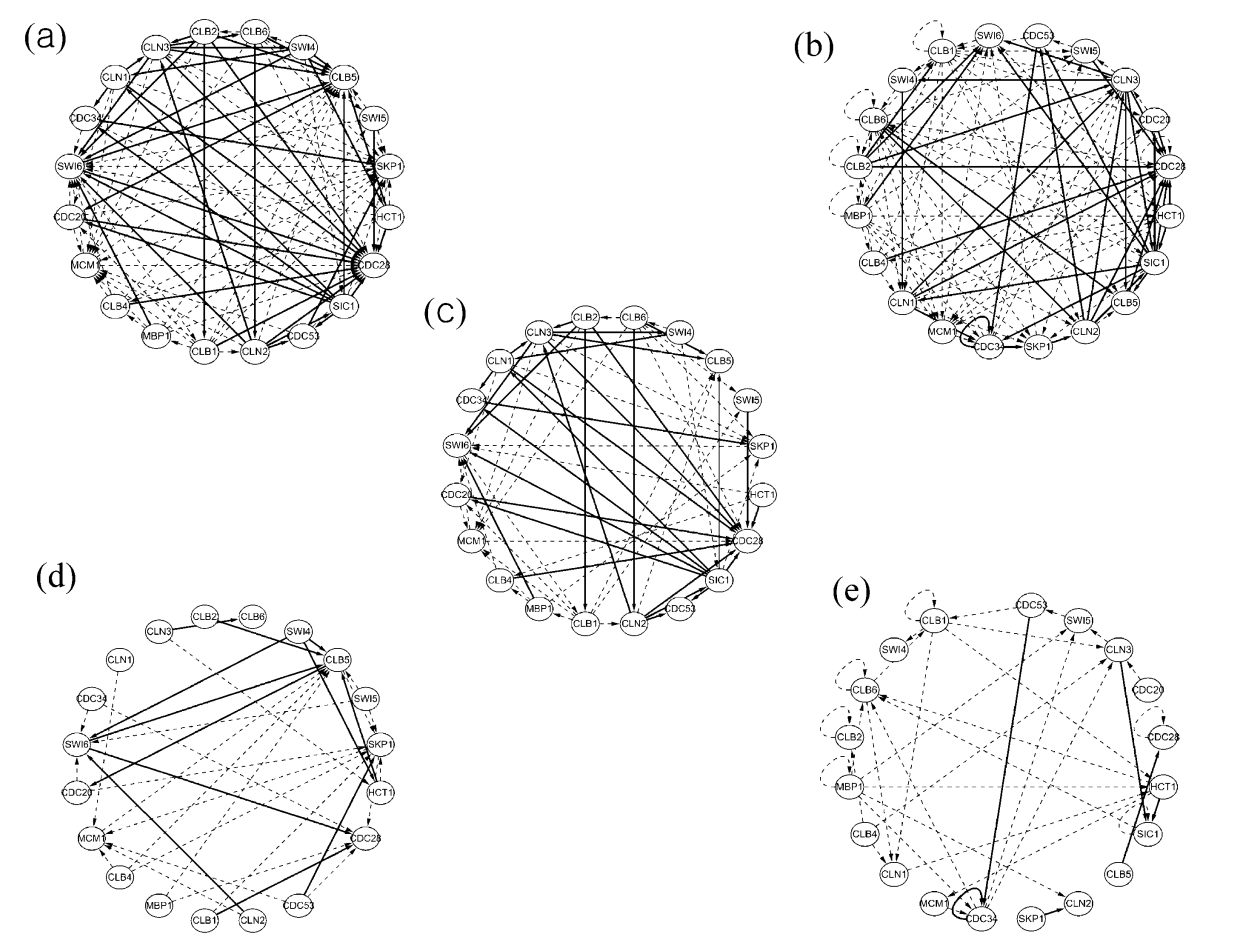
The inferred GRN by both BOLS and SBL are compared with the same expression data used in Table 2. (a) A GRN by BOLS, (b) A GRN by SBL, (c) The inferred interactions both by BOLS and SBL, (d) The inferred interactions only by BOLS, (e) The inferred interactions only by SBL. For all figures, the solid line correspond the inferred interactions which are identified as known physical or genetic interactions from the BioGRID database, and the dashed line the unknown interactions.

We also evaluate both BOLS and SBL using the same data set in Table 2 to compare the efficiency of BOLS with SBL. For direct comparison purpose, we make the graphs of inferred GRN by BOLS and SBL (Figure 7). There are 107 inferred interactions (45 of them are identified as known interactions from the database) by BOLS (Figure 7a) and 116 interactions (38 of them are known interactions) by SBL (Figure 7b). Figure 7c shows that 62 interactions are inferred by both BOLS and SBL, in which 32 of them are known interactions. Figure 7d shows 45 interactions only by BOLS in which 13 of them are identified as known interactions from the database. On the other hand, Figure 7e shows 54 interactions only by SBL in which only 6 of them are known interactions from the database. It is reasonable to assume that the complete information of biological interactions of Yeast is not available from the database yet. It is believed that the more depositions of the information concerning Yeast interactions are still on the way to reach the more complete understanding of the underlying complex cellular networks of Yeast. Based on the currently available information from the database, we can say that SBL algorithm infers GRN with relatively more complexity and less identified known interactions than BOLS. Therefore, based on our evaluation experiments with synthetic and Yeast expression data, it is sufficient to conclude that SBL produces more over-fitting solutions (i.e. more FP solutions) than BOLS.

## Conclusion

In the evaluation of BOLS using synthetic data, it is shown the proposed BOLS algorithm is able to reconstruct networks using a very limited number of experimental samples. In this study, we assume that there is significantly limited number of data points and the noise level in the data is not known. This is a common situation in expression data analysis. To handle these difficulties, we adopt a decomposition strategy to break down the entire network into a set of unit networks. This decomposition makes the inferring of a whole GRN into several separate regressions. Thus, if we have extremely small number of data points, our method can not provide 100% correct solutions, but provides each unit network with P(D|H_i_), which can be used as confidence level. The unit network with a higher P(D|H_i_) has a higher confidence such that the unit network is correctly inferred (Figure 5). Previously, Basso *et al. *[11] validated their ARACNe algorithm using 19 nodes synthetic network. With 350 sample size, the sensitivity and complementary specificity are approximately 80% and 20%, respectively. The inferred interactions from their method contain approximately 20% false positive and false negative interactions, respectively. With our method, it is possible to locate false positive or false negative interactions with P(D|H_i_)s, which is the unique property of BOLS algorithm. Our *in silico *experiment shows that the performance depends on the number of data points, genes, and noise level. Further study will be required to investigate the relationship between these parameters and the performance so that the BOLS algorithm can be generally applied to the microarray data with any number of genes, data points, and noise level.

Another evaluation is conducted with the Yeast *Saccharomyces cerevisiae *data of 20 genes, which are involved in cell-cycle regulation. In the output network, there is noticeable number of interactions that are identified as physical or genetic interactions from the literature. There are also several interactions that are not identified from the literature. However, it is shown that the definition of these genes from the BioGRID database gives enough evidences that some of them have indirect interactions. Thus, this experiment shows that BOLS algorithm is able to elucidate remarkable number of known physical or genetic events.

For both evaluation experiments with synthetic and Yeast expression data, we compare the performance between BOLS and SBL algorithms. The SBL algorithm [18,19,24] is a general Bayesian framework to obtain sparse solutions utilizing linear models. This method is known as *type-II maximum likelihood *method [24], in which the solutions are obtained by maximizing the marginal likelihood. On the other hand, BOLS utilizes the Bayesian model selection that is an extension of maximum likelihood model selection, in which the posterior is obtained by multiplying the best fit likelihood by the *Occam's factor*. From our both evaluation experiments, it is concluded that BOLS produces sparser solutions with less FP than SBL does.

## Methods

### 1. Gene regulatory network model and Unit networks

To study GRN, we choose a system of DBN as our GRN model. This model is described by

ei(t+1)=∑j=1Kwijej(t)+ξi(t) for t=1,2,…,N−1.
 MathType@MTEF@5@5@+=feaafiart1ev1aaatCvAUfKttLearuWrP9MDH5MBPbIqV92AaeXatLxBI9gBaebbnrfifHhDYfgasaacH8akY=wiFfYdH8Gipec8Eeeu0xXdbba9frFj0=OqFfea0dXdd9vqai=hGuQ8kuc9pgc9s8qqaq=dirpe0xb9q8qiLsFr0=vr0=vr0dc8meaabaqaciaacaGaaeqabaqabeGadaaakeaacqWGLbqzdaWgaaWcbaGaemyAaKgabeaakmaabmaabaGaemiDaqNaey4kaSIaeGymaedacaGLOaGaayzkaaGaeyypa0ZaaabCaeaacqWG3bWDdaWgaaWcbaGaemyAaKMaemOAaOgabeaakiabdwgaLnaaBaaaleaacqWGQbGAaeqaaOWaaeWaaeaacqWG0baDaiaawIcacaGLPaaaaSqaaiabdQgaQjabg2da9iabigdaXaqaaiabdUealbqdcqGHris5aOGaey4kaSccciGae8NVdG3aaSbaaSqaaiabdMgaPbqabaGcdaqadaqaaiabdsha0bGaayjkaiaawMcaaiabbccaGiabdAgaMjabd+gaVjabdkhaYjabbccaGiabdsha0jabg2da9iabigdaXiabcYcaSiabikdaYiabcYcaSiablAciljabcYcaSiabd6eaojabgkHiTiabigdaXiabc6caUaaa@5F54@

Here N is the number of data points, K is the number of gene in the data, and *ξ*_i_(t) is a noise at any time. The e_i_(t)s are the level of mRNAs at any given time t, which influences the expression levels of the gene. The value w_ij _describes the interaction strength between the j^th ^gene and the i^th ^gene. This model has the first order Markov property, which means that the future expression e_i_(t+1) is independent of the past expression level e_i_(t-1) given the present expression level e_i_(t). As briefly mentioned in the previous section, it is required that the data set is reorganized into a linear system to reverse engineer GRN using BOLS algorithm. This GRN model can be easily rewritten into a linear system as

*Y*_*i *_= *Ew*_*i *_+ *n*_*i*_

where i = 1, 2,..., K. *w*_*i *_is a column matrix of regulation strength values, which is defined as *w*_*i *_= [w_i1_, w_i2_,......, w_ik_]^T^. *Y*_*i *_is a column matrix of expression levels for target genes, which is defined as *Y*_*i *_= [e_i_(2), e_i_(3),..., e_i_(N)]^T^. *E *is a N-1 × K design matrix, which is defined as *E *= [e_1_, e_2_,......, e_K_] and e_i _= [e_i_(1), e_i_(2),......, e_i_(N-1)]^T^. *n*_*i *_is an Gaussian noises, which is defined as *n*_*i *_= [n_i_(1), n_i_(2),..., n_i_(N-1)]^T^.

It should be noted that the expression levels e_i_(t)s in both Eq. 1 and 2 are same and noisy ones. Eq. 1 describes that the current expression levels e_i_(t)s are determined depending on the previous ones e_i_(t-1) and noises *ξ*_i_(t-1) and the expression levels are evolved over the time based on the GRN. Hence, *ξ*_i_(t) is a noise added into e_i_(t)s during the generation of synthetic or real expression levels based on DBN model. Once the "noisy" expression levels are available, we consider Eq. 2 for reverse engineering of GRN. Because the given expression levels e_i_(t)s in Eq. 2 are noisy, we should have a condition such that |*Y*_*i *_- *Ew*_*i*_| = |*n*_*i*_| > 0. If *n*_*i *_= 0, we will have over-fitting solutions, in which model fitting oscillates widely so as to fit the noise. Thus, we can say that *n*_*i *_is a noise related with "data misfit" or "confidence interval" on the best fit parameters. On the other hand, *ξ*_i_(t) is a noise related with the "generation" of expression levels. If *n*_*i *_is modeled as zero-mean Gaussian noise with standard deviation *σ*_n_, the probability of the data given the parameter *w*_*i *_is

P(D|wi,β)=exp⁡(−βED(D|wi))ZD(β)
 MathType@MTEF@5@5@+=feaafiart1ev1aaatCvAUfKttLearuWrP9MDH5MBPbIqV92AaeXatLxBI9gBaebbnrfifHhDYfgasaacH8akY=wiFfYdH8Gipec8Eeeu0xXdbba9frFj0=OqFfea0dXdd9vqai=hGuQ8kuc9pgc9s8qqaq=dirpe0xb9q8qiLsFr0=vr0=vr0dc8meaabaqaciaacaGaaeqabaqabeGadaaakeaacqWGqbaudaqadaqaaiabdseaejabcYha8jabdEha3naaBaaaleaacqWGPbqAaeqaaOGaeiilaWccciGae8NSdigacaGLOaGaayzkaaGaeyypa0ZaaSaaaeaacyGGLbqzcqGG4baEcqGGWbaCdaqadaqaaiabgkHiTiab=j7aIjabdweafnaaBaaaleaacqWGebaraeqaaOWaaeWaaeaacqWGebarcqGG8baFcqWG3bWDdaWgaaWcbaGaemyAaKgabeaaaOGaayjkaiaawMcaaaGaayjkaiaawMcaaaqaaiabdQfaAnaaBaaaleaacqWGebaraeqaaOWaaeWaaeaacqWFYoGyaiaawIcacaGLPaaaaaaaaa@5000@

where *β *= 1/*σ*_n_^2^, E_D _= (Y_i_-Ew_i_)^T^(Y_i_-Ew_i_), and Z_D _= (2*π*/*β*)^N/2^. P(D|w_i_, *β*) is called the maximum likelihood. It is well known that maximum likelihood is *underdetermined *and *ill conditioned *problems. Thus, we are motivated to develop a novel strategy to overcome these problems.

We decompose the entire network into a set of small networks that are defined as unit networks. Each unit network consists of one particular target gene and its regulator genes. The unit network is used as input and output of BOLS algorithm. Figure 2a presents an input unit network that includes all genes in the data set as regulator candidates. Figure 2b presents an output unit network that contains most probable regulator genes to the target gene. Thus, we can decompose the GRN into several separate regressions and apply the BOLS algorithm to each unit network. Therefore, for GRN with K number of genes, we will run the algorithm K times-producing a unit network for each time. It should be noted that we can use all unit networks to create whole GRN that consists of two finite sets, a set of nodes (genes) and a set of edges (interactions) such that each edge connects two nodes. With all (or selected) unit networks, the generation of GRN can easily be generalized by constructing a *K *× *K graph matrix *G = [g_i, j_], with binary element

gi,j={1if gene i and gene j have regulatory relationship0otherwise.
 MathType@MTEF@5@5@+=feaafiart1ev1aaatCvAUfKttLearuWrP9MDH5MBPbIqV92AaeXatLxBI9gBaebbnrfifHhDYfgasaacH8akY=wiFfYdH8Gipec8Eeeu0xXdbba9frFj0=OqFfea0dXdd9vqai=hGuQ8kuc9pgc9s8qqaq=dirpe0xb9q8qiLsFr0=vr0=vr0dc8meaabaqaciaacaGaaeqabaqabeGadaaakeaacqWGNbWzdaWgaaWcbaGaemyAaKMaeiilaWIaemOAaOgabeaakiabg2da9maaceaabaqbaeaabiGaaaqaaiabigdaXaqaaiabdMgaPjabdAgaMjabbccaGiabdEgaNjabdwgaLjabd6gaUjabdwgaLjabbccaGiabdMgaPjabbccaGiabdggaHjabd6gaUjabdsgaKjabbccaGiabdEgaNjabdwgaLjabd6gaUjabdwgaLjabbccaGiabdQgaQjabbccaGiabdIgaOjabdggaHjabdAha2jabdwgaLjabbccaGiabdkhaYjabdwgaLjabdEgaNjabdwha1jabdYgaSjabdggaHjabdsha0jabd+gaVjabdkhaYjabdMha5jabbccaGiabdkhaYjabdwgaLjabdYgaSjabdggaHjabdsha0jabdMgaPjabd+gaVjabd6gaUjabdohaZjabdIgaOjabdMgaPjabdchaWbqaaiabicdaWaqaaiabd+gaVjabdsha0jabdIgaOjabdwgaLjabdkhaYjabdEha3jabdMgaPjabdohaZjabdwgaLjabc6caUaaaaiaawUhaaaaa@819E@

Furthermore, this matrix induces a GRN, in which nodes corresponds to genes and an edge joins nodes *i *and node *j *if and only if g_*i*, *j *_= 1. For each edge, we store the information of unit network where it belongs. The information of these sets can be easily obtainable from all unit networks. Thus, the combination of all unit networks for creating whole GRN is easy and straightforward procedure.

### 2. Bayesian orthogonal least square algorithm

As briefly described in background section, reverse engineering with a linear system with limited data has to overcome two difficulties. In this section, we describe our efforts to overcome these challenges by developing BOLS algorithm.

The system is referred as *underdetermined *when the number of parameters is larger than the number of available data points, so that standard least squares techniques break down. This issue can be solved with the OLS [22] method involves decomposition of the design matrix into two using Gram-Schmidt Orthogonalization theory as,

E = XU

where E_N-1 × K _= [e_1_, e_2_,......, e_k_], X_N-1 × K _= [x_1_, x_2_,......, x_k_] and U_K × K _is a triangular matrix with 1's on the diagonal and 0's below the diagonal, that is,

U=[1u12u13⋯u1k01u23u2k00⋱⋱⋮⋮⋱1uk−1k0⋯001].
 MathType@MTEF@5@5@+=feaafiart1ev1aaatCvAUfKttLearuWrP9MDH5MBPbIqV92AaeXatLxBI9gBaebbnrfifHhDYfgasaacH8akY=wiFfYdH8Gipec8Eeeu0xXdbba9frFj0=OqFfea0dXdd9vqai=hGuQ8kuc9pgc9s8qqaq=dirpe0xb9q8qiLsFr0=vr0=vr0dc8meaabaqaciaacaGaaeqabaqabeGadaaakeaacqWGvbqvcqGH9aqpdaWadaqaauaabeqafuaaaaaabaGaeGymaedabaGaemyDau3aaSbaaSqaaiabigdaXiabikdaYaqabaaakeaacqWG1bqDdaWgaaWcbaGaeGymaeJaeG4mamdabeaaaOqaaiabl+Uimbqaaiabdwha1naaBaaaleaacqaIXaqmcqWGRbWAaeqaaaGcbaGaeGimaadabaGaeGymaedabaGaemyDau3aaSbaaSqaaiabikdaYiabiodaZaqabaaakeaaaeaacqWG1bqDdaWgaaWcbaGaeGOmaiJaem4AaSgabeaaaOqaaiabicdaWaqaaiabicdaWaqaaiablgVipbqaaiablgVipbqaaiabl6Uinbqaaiabl6UinbqaaaqaaiablgVipbqaaiabigdaXaqaaiabdwha1naaBaaaleaacqWGRbWAcqGHsislcqaIXaqmcqWGRbWAaeqaaaGcbaGaeGimaadabaGaeS47IWeabaGaeGimaadabaGaeGimaadabaGaeGymaedaaaGaay5waiaaw2faaiabc6caUaaa@61A8@

Let's say that w is the regression parameter inferred by E and g is the regression parameter inferred by X. It is noted that g and w satisfy the triangular system

g = Uw.

The computational procedure of Gram-Schmidt method is described as

x1=e1uij=xiTej/(xiTxi),⤢⤢⤢1≤i≤jxj=ej−∑i=1K−1uijxi,⤢⤢⤢2≤j≤K.
 MathType@MTEF@5@5@+=feaafiart1ev1aaatCvAUfKttLearuWrP9MDH5MBPbIqV92AaeXatLxBI9gBaebbnrfifHhDYfgasaacH8akY=wiFfYdH8Gipec8Eeeu0xXdbba9frFj0=OqFfea0dXdd9vqai=hGuQ8kuc9pgc9s8qqaq=dirpe0xb9q8qiLsFr0=vr0=vr0dc8meaabaqaciaacaGaaeqabaqabeGadaaakqaabeqaaiabdIha4naaBaaaleaacqaIXaqmaeqaaOGaeyypa0Jaemyzau2aaSbaaSqaaiabigdaXaqabaaakeaacqWG1bqDdaWgaaWcbaGaemyAaKMaemOAaOgabeaakiabg2da9iabdIha4naaDaaaleaacqWGPbqAaeaacqWGubavaaGccqWGLbqzdaWgaaWcbaGaemOAaOgabeaakiabc+caVmaabmaabaGaemiEaG3aa0baaSqaaiabdMgaPbqaaiabdsfaubaakiabdIha4naaBaaaleaacqWGPbqAaeqaaaGccaGLOaGaayzkaaGaeiilaWIaaGzbRlaaywW6caaMfSUaeGymaeJaeyizImQaemyAaKMaeyizImQaemOAaOgabaGaemiEaG3aaSbaaSqaaiabdQgaQbqabaGccqGH9aqpcqWGLbqzdaWgaaWcbaGaemOAaOgabeaakiabgkHiTmaaqahabaGaemyDau3aaSbaaSqaaiabdMgaPjabdQgaQbqabaGccqWG4baEdaWgaaWcbaGaemyAaKgabeaaaeaacqWGPbqAcqGH9aqpcqaIXaqmaeaacqWGlbWscqGHsislcqaIXaqma0GaeyyeIuoakiabcYcaSiaaywW6caaMfSUaaGzbRlabikdaYiabgsMiJkabdQgaQjabgsMiJkabdUealjabc6caUaaaaa@7AAE@

Because x_i _and x_j _(i ≠ j) are orthogonal to each other, the sum of square of Y is defined as

YTY=(xiTxi)gi2+niTni.
 MathType@MTEF@5@5@+=feaafiart1ev1aaatCvAUfKttLearuWrP9MDH5MBPbIqV92AaeXatLxBI9gBaebbnrfifHhDYfgasaacH8akY=wiFfYdH8Gipec8Eeeu0xXdbba9frFj0=OqFfea0dXdd9vqai=hGuQ8kuc9pgc9s8qqaq=dirpe0xb9q8qiLsFr0=vr0=vr0dc8meaabaqaciaacaGaaeqabaqabeGadaaakeaacqWGzbqwdaahaaWcbeqaaiabdsfaubaakiabdMfazjabg2da9maabmaabaGaemiEaG3aa0baaSqaaiabdMgaPbqaaiabdsfaubaakiabdIha4naaBaaaleaacqWGPbqAaeqaaaGccaGLOaGaayzkaaGaem4zaC2aa0baaSqaaiabdMgaPbqaaiabikdaYaaakiabgUcaRiabd6gaUnaaDaaaleaacqWGPbqAaeaacqWGubavaaGccqWGUbGBdaWgaaWcbaGaemyAaKgabeaakiabc6caUaaa@471E@

The variance of Y_i _is defined as

YTY/N−1=(xiTxi)gi2/N−1+niTni/N−1.
 MathType@MTEF@5@5@+=feaafiart1ev1aaatCvAUfKttLearuWrP9MDH5MBPbIqV92AaeXatLxBI9gBaebbnrfifHhDYfgasaacH8akY=wiFfYdH8Gipec8Eeeu0xXdbba9frFj0=OqFfea0dXdd9vqai=hGuQ8kuc9pgc9s8qqaq=dirpe0xb9q8qiLsFr0=vr0=vr0dc8meaabaqaciaacaGaaeqabaqabeGadaaakeaadaWcgaqaaiabdMfaznaaCaaaleqabaGaemivaqfaaOGaemywaKfabaGaemOta4KaeyOeI0IaeGymaedaaiabg2da9maalyaabaWaaeWaaeaacqWG4baEdaqhaaWcbaGaemyAaKgabaGaemivaqfaaOGaemiEaG3aaSbaaSqaaiabdMgaPbqabaaakiaawIcacaGLPaaacqWGNbWzdaqhaaWcbaGaemyAaKgabaGaeGOmaidaaaGcbaGaemOta4KaeyOeI0IaeGymaedaaiabgUcaRmaalyaabaGaemOBa42aa0baaSqaaiabdMgaPbqaaiabdsfaubaakiabd6gaUnaaBaaaleaacqWGPbqAaeqaaaGcbaGaemOta4KaeyOeI0IaeGymaedaaiabc6caUaaa@5066@

It is noticed that (x_i_^T^x_i_)g_i_^2^/N-1 is the variance of Y_i _which is contributed by the regressors and n_i_^T^n_i_/N-1 is the noise (or unexplained) variance of Y_i_. Hence, (x_i_^T^x_i_)g_i_^2^/N-1 is the increment to the variance of Y_i _contributed by w_I_, and the error reduction ratio only due to x_i _can be defined as

[NError]_i _= (x_i_^T^x_i_)g_i_^2^/(Y^T^Y), 1 ≤ i ≤ K.

This *error *term provides a simple and efficient measure for seeking a subset of significant regression parameters in a forward-regression way. The repressor selection procedure from Chen *et al.*[22] is summarized as follows:

1) At the first step, for 1 ≤ i < K, compute

x_1_^(i) ^= e_i_

g_1_^(i) ^= (x_1_^(i)^)^T^Y/((x_1_^(i)^)^Tx^_1_^(i)^)

[NError]_1_^(i) ^= (g_1_^(i)^)^2^(x_1_^(i)T^x_1_^(i)^)/(Y^T^Y)

Find

[NError]_1_^(i1) ^= max{[NError]_1_^(i)^, 1 ≤ i ≤ K}

and select

x_1 _= x_1_^(i1) ^= e_i1_.

(2) At the j^th ^step where j ≥ 2, for 1 ≤ i ≤ K, i ≠ i_1_,..., i ≠ = i_j-1_, compute

u_mj_^(i) ^= x_m_^T^e_i_/(x_m_^T^x_l_), 1 ≤ m < j

xj(i)=ei−∑m=1j−1umj(i)xm
 MathType@MTEF@5@5@+=feaafiart1ev1aaatCvAUfKttLearuWrP9MDH5MBPbIqV92AaeXatLxBI9gBamXvP5wqSXMqHnxAJn0BKvguHDwzZbqegyvzYrwyUfgarqqtubsr4rNCHbGeaGqiA8vkIkVAFgIELiFeLkFeLk=iY=Hhbbf9v8qqaqFr0xc9pk0xbba9q8WqFfeaY=biLkVcLq=JHqVepeea0=as0db9vqpepesP0xe9Fve9Fve9GapdbaqaaeGacaGaaiaabeqaamqadiabaaGcbaGaeeiEaG3aa0baaSqaaiabbQgaQbqaaiabcIcaOiabbMgaPjabcMcaPaaakiabg2da9iabbwgaLnaaBaaaleaacqqGPbqAaeqaaOGaeyOeI0YaaabCaeaacqWG1bqDdaqhaaWcbaGaemyBa0MaemOAaOgabaGaeiikaGIaemyAaKMaeiykaKcaaOGaemiEaG3aaSbaaSqaaiabd2gaTbqabaaabaGaemyBa0Maeyypa0JaeGymaedabaGaemOAaOMaeyOeI0IaeGymaedaniabggHiLdaaaa@5B1B@

g_j_^(i) ^= (x_j_^(i)^)^T^Y/((x_j_^(i)^)^T^x_j_^(i)^)

[NError]_j_^(i) ^= (g_j_^(i)^)^2^(x_j_^(i)^)^T^x_j_^(i)^/(Y^T^Y)

Find

[NError]_j_^(ij) ^= max{[NError]_j_^(i)^, 1 ≤ i ≤ K, i ≠ i_1_,..., i ≠ = i_j-1 _}

and select

xj=xj(ii)=eij−∑m=1j−1umj(i)xm,
 MathType@MTEF@5@5@+=feaafiart1ev1aaatCvAUfKttLearuWrP9MDH5MBPbIqV92AaeXatLxBI9gBamXvP5wqSXMqHnxAJn0BKvguHDwzZbqegyvzYrwyUfgarqqtubsr4rNCHbGeaGqiA8vkIkVAFgIELiFeLkFeLk=iY=Hhbbf9v8qqaqFr0xc9pk0xbba9q8WqFfeaY=biLkVcLq=JHqVepeea0=as0db9vqpepesP0xe9Fve9Fve9GapdbaqaaeGacaGaaiaabeqaamqadiabaaGcbaGaeeiEaG3aaSbaaSqaaiabbQgaQbqabaGccqGH9aqpcqqG4baEdaqhaaWcbaGaeeOAaOgabaGaeiikaGIaeeyAaKMaeeyAaKMaeiykaKcaaOGaeyypa0Jaeeyzau2aaSbaaSqaaiabbMgaPjabbQgaQbqabaGccqGHsisldaaeWbqaaiabdwha1naaDaaaleaacqWGTbqBcqWGQbGAaeaacqGGOaakcqWGPbqAcqGGPaqkaaGccqWG4baEdaWgaaWcbaGaemyBa0gabeaaaeaacqWGTbqBcqGH9aqpcqaIXaqmaeaacqWGQbGAcqGHsislcqaIXaqma0GaeyyeIuoakiabcYcaSaaa@62C7@

where u_mj _= u_mj_^(ij)^, 1 ≤ m < j.

(3) The OLS is terminated at the K_s_^th ^step when

1−∑m=1KS[NError]m<ρ,
 MathType@MTEF@5@5@+=feaafiart1ev1aaatCvAUfKttLearuWrP9MDH5MBPbIqV92AaeXatLxBI9gBamXvP5wqSXMqHnxAJn0BKvguHDwzZbqegyvzYrwyUfgarqqtubsr4rNCHbGeaGqiA8vkIkVAFgIELiFeLkFeLk=iY=Hhbbf9v8qqaqFr0xc9pk0xbba9q8WqFfeaY=biLkVcLq=JHqVepeea0=as0db9vqpepesP0xe9Fve9Fve9GapdbaqaaeGacaGaaiaabeqaamqadiabaaGcbaGaeGymaeJaeyOeI0YaaabCaeaadaWadaqaaiabd6eaojabdweafjabdkhaYjabdkhaYjabd+gaVjabdkhaYbGaay5waiaaw2faamaaBaaaleaacqWGTbqBaeqaaOGaeyipaWdcciGae8xWdihaleaacqWGTbqBcqGH9aqpcqaIXaqmaeaacqWGlbWsdaWgaaadbaGaem4uamfabeaaa0GaeyyeIuoakiabcYcaSaaa@5604@

where 0 <*ρ *< 1 is a chosen tolerance.

We assume that we do not have any information about noise level in the data, so that we completely over-fit the data to the model using OLS method with *ρ *<< 1 (*ρ *= 1.0e-3 in this study). Then we reduce unnecessary parameters to deal with the *ill-conditioned *problem by using second order derivative for network pruning techniques and Bayesian model comparison framework. We can obtain the optimal solution by trading off between the complexity of the model and the data misfit [20]. We start this procedure using an extremely small value for data misfit by completely over-fitting the data to the model. As the complexity of the model is reduced; i.e. the number of effective parameters is reduced, the value for data misfit is increased. The optimal complexity of the model for "true solution" is decided using a Bayesian model comparison frame that assigns a preference to the model H_i _with certain complexity, a Bayesian evaluation so called as the evidence P(D|H_i_). The evidence is obtained by multiplying the best-fit likelihood by the *Occam's factor*,

P(D|Hi)=∫P(D|gi,Hi)P(gi|Hi)dg≅P(D|gMP,Hi)P(gMP|Hi)(2π)k/2det⁡−1/2A
 MathType@MTEF@5@5@+=feaafiart1ev1aaatCvAUfKttLearuWrP9MDH5MBPbIqV92AaeXatLxBI9gBaebbnrfifHhDYfgasaacH8akY=wiFfYdH8Gipec8Eeeu0xXdbba9frFj0=OqFfea0dXdd9vqai=hGuQ8kuc9pgc9s8qqaq=dirpe0xb9q8qiLsFr0=vr0=vr0dc8meaabaqaciaacaGaaeqabaqabeGadaaakeaafaqadeGacaaabaGaemiuaa1aaeWaaeaacqWGebarcqGG8baFcqWGibasdaWgaaWcbaGaemyAaKgabeaaaOGaayjkaiaawMcaaaqaaiabg2da9maapeaabaGaemiuaa1aaeWaaeaacqWGebarcqGG8baFcqWGNbWzdaWgaaWcbaGaemyAaKgabeaakiabcYcaSiabdIeainaaBaaaleaacqWGPbqAaeqaaaGccaGLOaGaayzkaaGaemiuaa1aaeWaaeaacqWGNbWzdaWgaaWcbaGaemyAaKgabeaakiabcYha8jabdIeainaaBaaaleaacqWGPbqAaeqaaaGccaGLOaGaayzkaaGaemizaqMaem4zaCgaleqabeqdcqGHRiI8aaGcbaaabaGaeyyrIaKaemiuaa1aaeWaaeaacqWGebarcqGG8baFcqWGNbWzdaWgaaWcbaGaemyta0KaemiuaafabeaakiabcYcaSiabdIeainaaBaaaleaacqWGPbqAaeqaaaGccaGLOaGaayzkaaGaemiuaa1aaeWaaeaacqWGNbWzdaWgaaWcbaGaemyta0KaemiuaafabeaakiabcYha8jabdIeainaaBaaaleaacqWGPbqAaeqaaaGccaGLOaGaayzkaaWaaeWaaeaacqaIYaGmiiGacqWFapaCaiaawIcacaGLPaaadaahaaWcbeqaaiabdUgaRjabc+caViabikdaYaaakiGbcsgaKjabcwgaLjabcsha0naaCaaaleqabaGaeyOeI0IaeGymaeJaei4la8IaeGOmaidaaOGaemyqaeeaaaaa@791D@

where P(D|g_*MP*_, H_i_) corresponds to the *best fit likelihood*, P(g_*MP*_|H_i_)(2*π*)^K/2^det^-1/2^A corresponds to the *Occam's factor*, A = ∂^2^logP(g|D, Hi)/∂g^2^, and g_*MP *_represents the most probable parameters of g. The *Occam's factor *is equal to the ratio of the posterior accessible volume of H_i_'s parameter space to the prior accessible volume, or the factor by which H_i_'s hypothesis space collapses when the data is collected [20]. The model H_i_s can be viewed as consisting of a certain number of exclusive sub-models, of which only one is chosen when the data is collected. The *Occam's factor *is a measure of complexity of the model that depends not only on the number of parameters in the model but also on the prior probability of the model. Therefore, the over-fitting solution can be avoided by using Bayesian model comparison frame because the Bayesian *Occam's factor *assures getting the optimal complexity of the model. See Mackay [20] for more details of *Occam's factor*.

With a second order derivative for network pruning [23], we can select the parameters to be eliminated first. Our goal here is to find a set of parameters whose deletion causes the least increase of cost function C

C = *β*/2(Y - Xg)^T ^(Y - Xg) + *α*/2(g^T ^g),

where *α *and *β *are hyper-parameters that measure the complexity of the model and data misfit, respectively. It will be shown later in this section the iterative formulae to estimate *α *and *β *with a given data set and model structure in Eq. 6 and 7. Using the second order derivative for network pruning method, we can derive the saliency equation as follows,

Lj=12(gj2Ajj−1)−α(gjAjj−1)(gTA−1vj)−α22[gTA−1g−(gTA−1vj)2Ajj−1]
 MathType@MTEF@5@5@+=feaafiart1ev1aaatCvAUfKttLearuWrP9MDH5MBPbIqV92AaeXatLxBI9gBaebbnrfifHhDYfgasaacH8akY=wiFfYdH8Gipec8Eeeu0xXdbba9frFj0=OqFfea0dXdd9vqai=hGuQ8kuc9pgc9s8qqaq=dirpe0xb9q8qiLsFr0=vr0=vr0dc8meaabaqaciaacaGaaeqabaqabeGadaaakeaacqWGmbatdaWgaaWcbaGaemOAaOgabeaakiabg2da9maalaaabaGaeGymaedabaGaeGOmaidaamaabmaabaWaaSaaaeaacqWGNbWzdaqhaaWcbaGaemOAaOgabaGaeGOmaidaaaGcbaGaemyqae0aa0baaSqaaiabdQgaQjabdQgaQbqaaiabgkHiTiabigdaXaaaaaaakiaawIcacaGLPaaacqGHsisliiGacqWFXoqydaqadaqaamaalaaabaGaem4zaC2aaSbaaSqaaiabdQgaQbqabaaakeaacqWGbbqqdaqhaaWcbaGaemOAaOMaemOAaOgabaGaeyOeI0IaeGymaedaaaaaaOGaayjkaiaawMcaamaabmaabaGaem4zaC2aaWbaaSqabeaacqWGubavaaGccqWGbbqqdaahaaWcbeqaaiabgkHiTiabigdaXaaakiabdAha2naaBaaaleaacqWGQbGAaeqaaaGccaGLOaGaayzkaaGaeyOeI0YaaSaaaeaacqWFXoqydaahaaWcbeqaaiabikdaYaaaaOqaaiabikdaYaaadaWadaqaaiabdEgaNnaaCaaaleqabaGaemivaqfaaOGaemyqae0aaWbaaSqabeaacqGHsislcqaIXaqmaaGccqWGNbWzcqGHsisldaWcaaqaamaabmaabaGaem4zaC2aaWbaaSqabeaacqWGubavaaGccqWGbbqqdaahaaWcbeqaaiabgkHiTiabigdaXaaakiabdAha2naaBaaaleaacqWGQbGAaeqaaaGccaGLOaGaayzkaaWaaWbaaSqabeaacqaIYaGmaaaakeaacqWGbbqqdaqhaaWcbaGaemOAaOMaemOAaOgabaGaeyOeI0IaeGymaedaaaaaaOGaay5waiaaw2faaaaa@754C@

where A_jj-_^1 ^is a j^th ^diagonal component of the inverse matrix of A, A = ∂^2^C/∂**g**^2^, and v_j _is the unit vector in parameter space, the j^th ^dimension at which it is equal to one and the rest of the dimensions are equal to zero. It should be noted that A and A^-1 ^are diagonal matrices because of the decomposition of design matrix E by Gram-Schmidt Orthogonalization theory. With Eq. 3 we can select parameters, whose elimination produces the least increase of cost function C.

With a Bayesian frame [20] we can compare alternative models when our model structures keep changing with the network pruning method. Let's say we have a data set D = [Y, X], where Y = [y(1), y(2),..., y(N)]^T ^is the target data set, X = [x_1_, x_2_,..., x_K_] the *N *× *K design matrix*, and x_i _= [x_i_(1), x_i_(2),..., x_i_(N)]^T ^each column matrix in X. The regression parameters we want to infer are g = [g_1_, g_2_,..., g_K_]^T^. The log posterior probability of data D, given *α *and *β*, can be derived [20] as,

log⁡(P(D|α,β,Hi))=−12β(Y−XgMP)T(Y−XgMP)−12αgMPTgMP−12log⁡|A|+k2log⁡(α)+N2log⁡(β)−N2log⁡(2π)
 MathType@MTEF@5@5@+=feaafiart1ev1aaatCvAUfKttLearuWrP9MDH5MBPbIqV92AaeXatLxBI9gBamXvP5wqSXMqHnxAJn0BKvguHDwzZbqegyvzYrwyUfgarqqtubsr4rNCHbGeaGqiA8vkIkVAFgIELiFeLkFeLk=iY=Hhbbf9v8qqaqFr0xc9pk0xbba9q8WqFfeaY=biLkVcLq=JHqVepeea0=as0db9vqpepesP0xe9Fve9Fve9GapdbaqaaeGacaGaaiaabeqaamqadiabaaGcbaqbaeaabiGaaaqaaiGbcYgaSjabc+gaVjabcEgaNnaabmaabaGaemiuaa1aaeWaaeaacqWGebarcqGG8baFiiGacqWFXoqycqGGSaalcqWFYoGycqGGSaalcqWGibasdaWgaaWcbaGaemyAaKgabeaaaOGaayjkaiaawMcaaaGaayjkaiaawMcaaiabg2da9aqaaiabgkHiTmaalaaabaGaeGymaedabaGaeGOmaidaaiab=j7aInaabmaabaGaemywaKLaeyOeI0IaemiwaGLaem4zaC2aaSbaaSqaaiabd2eanjabdcfaqbqabaaakiaawIcacaGLPaaadaahaaWcbeqaaiabdsfaubaakmaabmaabaGaemywaKLaeyOeI0IaemiwaGLaem4zaC2aaSbaaSqaaiabd2eanjabdcfaqbqabaaakiaawIcacaGLPaaacqGHsisldaWcaaqaaiabigdaXaqaaiabikdaYaaacqWFXoqycqWGNbWzdaqhaaWcbaGaemyta0KaemiuaafabaGaemivaqfaaOGaem4zaC2aaSbaaSqaaiabd2eanjabdcfaqbqabaaakeaaaeaacqGHsisldaWcaaqaaiabigdaXaqaaiabikdaYaaacyGGSbaBcqGGVbWBcqGGNbWzdaabdaqaaiabdgeabbGaay5bSlaawIa7aiabgUcaRmaalaaabaGaem4AaSgabaGaeGOmaidaaiGbcYgaSjabc+gaVjabcEgaNnaabmaabaGae8xSdegacaGLOaGaayzkaaGaey4kaSYaaSaaaeaacqWGobGtaeaacqaIYaGmaaGagiiBaWMaei4Ba8Maei4zaC2aaeWaaeaacqWFYoGyaiaawIcacaGLPaaacqGHsisldaWcaaqaaiabd6eaobqaaiabikdaYaaacyGGSbaBcqGGVbWBcqGGNbWzdaqadaqaaiabikdaYiab=b8aWbGaayjkaiaawMcaaaaaaaa@A0BB@

where the subscript MP denotes *Most Probable*. The evidence P(D|H_i_) can be obtained if we marginalize the probability defined in Eq. 5 over the hyper-parameters *α *and *β*. Before the estimation of the evidence P(D|H_i_), we have to find the *most probable *value of the hyper-parameters *α *and *β*. The differentiation of Eq. 5 over *α *and *β *and the rearrangement gives formulae for the iterative re-estimation of *α *and *β *[20],

α^:=γgTg
 MathType@MTEF@5@5@+=feaafiart1ev1aaatCvAUfKttLearuWrP9MDH5MBPbIqV92AaeXatLxBI9gBaebbnrfifHhDYfgasaacH8akY=wiFfYdH8Gipec8Eeeu0xXdbba9frFj0=OqFfea0dXdd9vqai=hGuQ8kuc9pgc9s8qqaq=dirpe0xb9q8qiLsFr0=vr0=vr0dc8meaabaqaciaacaGaaeqabaqabeGadaaakeaaiiGacuWFXoqygaqcaiabcQda6iabg2da9maalaaabaGae83SdCgabaGaem4zaC2aaWbaaSqabeaacqWGubavaaGccqWGNbWzaaaaaa@362C@

β^:=K−γ(Y−Xg)T(Y−Xg)
 MathType@MTEF@5@5@+=feaafiart1ev1aaatCvAUfKttLearuWrP9MDH5MBPbIqV92AaeXatLxBI9gBaebbnrfifHhDYfgasaacH8akY=wiFfYdH8Gipec8Eeeu0xXdbba9frFj0=OqFfea0dXdd9vqai=hGuQ8kuc9pgc9s8qqaq=dirpe0xb9q8qiLsFr0=vr0=vr0dc8meaabaqaciaacaGaaeqabaqabeGadaaakeaaiiGacuWFYoGygaqcaiabcQda6iabg2da9maalaaabaGaem4saSKaeyOeI0Iae83SdCgabaWaaeWaaeaacqWGzbqwcqGHsislcqWGybawcqWGNbWzaiaawIcacaGLPaaadaahaaWcbeqaaiabdsfaubaakmaabmaabaGaemywaKLaeyOeI0IaemiwaGLaem4zaCgacaGLOaGaayzkaaaaaaaa@420E@

where *γ *= N - *αTrace*(A^-1^), *g *= *A*^-1^*X*^T^*Y*, N is number of data points, and K is number of variables (genes). To rank alternative structures (or complexities) of the model in the light of data set D, we evaluate the evidence by marginalizing the posterior probability P(D|*α*, *β*, H_i_) over *α *and *β*,

*P*(*D*|*H*_i_) = ∫∫*P*(*D*|*α*, *β*, *H*_i_)*P*(*α*, *β*)*dαdβ*.

We have very little prior information about *α *and *β*. When the available prior information is minimal, the learning process is often started with an objective prior probability. This uninformative prior probability is referred to as "vague prior" for a parameter with a range from 0 to ∞, which is a flat prior [87]. This prior probability can be left out when we compare alternative models. With the prior available, we can marginalize the posterior P(D|*α*, *β*, H_i_). The marginalization of P(D|*α*, *β*, H_i_) over *α *and *β *can be estimated using a flat prior and Gaussian integration [20],

P(D|Hi)≅P(D|α^,β^,Hi)⋅P(log⁡α^,log⁡β^)⋅2π⋅σlog⁡α|D⋅2π⋅σlog⁡β|D∝P(D|α^,β^,Hi)⋅2π⋅σlog⁡α|D⋅2π⋅σlog⁡β|D,
 MathType@MTEF@5@5@+=feaafiart1ev1aaatCvAUfKttLearuWrP9MDH5MBPbIqV92AaeXatLxBI9gBaebbnrfifHhDYfgasaacH8akY=wiFfYdH8Gipec8Eeeu0xXdbba9frFj0=OqFfea0dXdd9vqai=hGuQ8kuc9pgc9s8qqaq=dirpe0xb9q8qiLsFr0=vr0=vr0dc8meaabaqaciaacaGaaeqabaqabeGadaaakeaafaqaaeGacaaabaGaemiuaa1aaeWaaeaacqWGebarcqGG8baFcqWGibasdaWgaaWcbaGaemyAaKgabeaaaOGaayjkaiaawMcaaaqaaiabgwKiajabdcfaqnaabmaabaGaemiraqKaeiiFaWhcciGaf8xSdeMbaKaacqGGSaalcuWFYoGygaqcaiabcYcaSiabdIeainaaBaaaleaacqWGPbqAaeqaaaGccaGLOaGaayzkaaGaeyyXICTaemiuaa1aaeWaaeaacyGGSbaBcqGGVbWBcqGGNbWzcuWFXoqygaqcaiabcYcaSiGbcYgaSjabc+gaVjabcEgaNjqb=j7aIzaajaaacaGLOaGaayzkaaGaeyyXIC9aaOaaaeaacqaIYaGmcqWFapaCcqGHflY1aSqabaGccqWFdpWCdaWgaaWcbaGagiiBaWMaei4Ba8Maei4zaCMae8xSdeMaeiiFaWNaemiraqeabeaakiabgwSixpaakaaabaGaeGOmaiJae8hWdahaleqaaOGaeyyXICTae83Wdm3aaSbaaSqaaiGbcYgaSjabc+gaVjabcEgaNjab=j7aIjabcYha8jabdseaebqabaaakeaaaeaacqGHDisTcqWGqbaudaqadaqaaiabdseaejabcYha8jqb=f7aHzaajaGaeiilaWIaf8NSdiMbaKaacqGGSaalcqWGibasdaWgaaWcbaGaemyAaKgabeaaaOGaayjkaiaawMcaaiabgwSixpaakaaabaGaeGOmaiJae8hWdaNaeyyXICnaleqaaOGae83Wdm3aaSbaaSqaaiGbcYgaSjabc+gaVjabcEgaNjab=f7aHjabcYha8jabdseaebqabaGccqGHflY1daGcaaqaaiabikdaYiab=b8aWbWcbeaakiabgwSixlab=n8aZnaaBaaaleaacyGGSbaBcqGGVbWBcqGGNbWzcqWFYoGycqGG8baFcqWGebaraeqaaOGaeiilaWcaaaaa@AA3F@

where *σ*_log*α*|D _and *σ*_log*β*|D _are the error bars on log*α *and log*β*, found by differentiating Eq. 5 twice:

σlog⁡α|D2≅2γ,
 MathType@MTEF@5@5@+=feaafiart1ev1aaatCvAUfKttLearuWrP9MDH5MBPbIqV92AaeXatLxBI9gBaebbnrfifHhDYfgasaacH8akY=wiFfYdH8Gipec8Eeeu0xXdbba9frFj0=OqFfea0dXdd9vqai=hGuQ8kuc9pgc9s8qqaq=dirpe0xb9q8qiLsFr0=vr0=vr0dc8meaabaqaciaacaGaaeqabaqabeGadaaakeaaiiGacqWFdpWCdaqhaaWcbaGagiiBaWMaei4Ba8Maei4zaCMae8xSdeMaeiiFaWNaemiraqeabaGaeGOmaidaaOGaeyyrIa0aaSaaaeaacqaIYaGmaeaacqWFZoWzaaGaeiilaWcaaa@3C9F@

σlog⁡β|D2≅2(N−γ).
 MathType@MTEF@5@5@+=feaafiart1ev1aaatCvAUfKttLearuWrP9MDH5MBPbIqV92AaeXatLxBI9gBaebbnrfifHhDYfgasaacH8akY=wiFfYdH8Gipec8Eeeu0xXdbba9frFj0=OqFfea0dXdd9vqai=hGuQ8kuc9pgc9s8qqaq=dirpe0xb9q8qiLsFr0=vr0=vr0dc8meaabaqaciaacaGaaeqabaqabeGadaaakeaaiiGacqWFdpWCdaqhaaWcbaGagiiBaWMaei4Ba8Maei4zaCMae8NSdiMaeiiFaWNaemiraqeabaGaeGOmaidaaOGaeyyrIa0aaSaaaeaacqaIYaGmaeaadaqadaqaaiabd6eaojabgkHiTiab=n7aNbGaayjkaiaawMcaaaaacqGGUaGlaaa@4040@

In this study, we create a novel reverse engineering algorithm for linear systems with K number of genes using three techniques described above. The algorithm is run K times so that all genes in the data set are considered as a target gene at least once. The algorithm of BOLS for a unit-network construction is summarized as

1. Set certain gene as the target gene and set remaining genes as regulator candidates as input.

2. Over-fit the data to Eq. 2 using OLS.

3. While the number of parameters is greater than 1.

3.1 Estimate *α *and *β *with iterative re-estimation Eq. 6 and 7.

3.2 Compute the P(D|H_i_) for the current state network H_i _with Eq. 8.

3.3. Find the parameter g_j _that gives the smallest L_j _by Eq. 4 and delete g_j_

4. Select the network with the maximum P(D|H_i_) as an output unit network.

## Authors' contributions

CSK developed BOLS algorithm, performed experiments for evaluation of BOLS algorithm, and drafted and finalized manuscript.

## Supplementary Material

Additional file 1 Comparing the performance between BOLS and SBL using the data set generated based on Rogers and Girolami's study – *Supplementary Information*. This description provides the comparison of performance between BOLS and SBL using the synthetic data generated by Rogers and Girolami [19]. Click here for file
