# Supplementary material for: Bayesian Orthogonal Least Squares (BOLS) algorithm for reverse engineering of gene regulatory networks
Source: BMC Bioinformatics. 2007 Jul 13;8:251. doi: 10.1186/1471-2105-8-251 (PMC1959566; doi:10.1186/1471-2105-8-251)
Supplement: Additional file 1 — Comparing the performance between BOLS and SBL using the data set generated based on Rogers and Girolami's study – Supplementary Information. This description provides the comparison of performance between BOLS and SBL using the synthetic data generated by Rogers and Girolami [19]. [file 1471-2105-8-251-S1.doc]

Comparing the performance between BOLS and SBL using the data set generated based on Rogers and Girolami’s study – *Supplementary Information*

*Chang Sik Kim*

*Bioinformatics Group, Turku Centre for Computer Science, Turku, Finland*

Email:cskim@kangwon.ac.kr

Tel: +82-10-6380-5670

In this supplementary note, we compare the performance between BOLS and SBL using the data that are generated based on Rogers and Girolami’s study [1]. They simulate knock-out experiments by numerically integrating a system of coupled nonlinear stochastic differential equations,

where the standard rate of degradation κ is set to 0.5, and λ is set to 0.01. A_+_ and A_ correspond to the positive (excitation) and negative (inhibitory) components of the *gene regulatory networks* matrix. The coefficient γ and β control the order of dependency between genes that have regulation relationships and *n* is an isotropic Gaussian noise term which is independent over time and has standard deviation ε. Given a set of initial conditions (in their case, these are set to zeros) and a step size (δt = 0.01), they simulate the expression levels for each gene i

,

where n is a vector of samples from an isotropic Gaussian with standard deviation ε and is scaled by the time step (to do this they multiply the noise by square root of the time step) to insure invariability of noise levels across different size of time steps. Gene knock-outs are simulated by forcing the expression of a particular gene to 0 throughout the course of simulation. This makes possible to take R replicates of any given gene knockout for both wild-type and mutants. The result of this data generation is 2R expression levels for each gene in each knock-out (mutant) system. In a network of K genes, this produces 2RK data points of expression levels. We use the Matlab code for the generations of synthetic networks and data from Roger and Girolami [1], which is available from http://www.dcs.gla.ac.uk/~srogers/reg_nets.html. For “fair” comparison, we compare the performance between the BOLS method and the SBL method by Tipping [2] using the data set generated by Rogers and Girolami. The matlab code of SBL by Tipping [2] is available from http://research.microsoft.com/mlp/RVM/default.htm. ROC comparison of BOLS and SBL output with K = 30 are shown in Figure S1. Note that ROC calculations are done based on Rogers and Girolami’s [1] study.

In Figure S1, it is shown that the overall results of BOLS and SBL are relatively lower than the ones with data sets generated based on DBN (Table 1). Obviously, it might be reasonable to have the difference in the results, because these data sets are generated by two different models, i.e. “nonlinear” differential model and DBN model. It should be also noted that BOLS produces relatively lower complementary specificity than SBL algorithm. Thus, it can be concluded that BOLS produces the solutions with less FP than SBL using the data set generated based on Rogers and Girolami’s [1] knock out experiment.

### Reference

1. Rogers S, Girolami, M: **A Bayesian regression approach to the inference of regulatory networks from gene expression data.** *Bioinformatics* 2005, **21**:3131-3137.

2. Tipping ME: **Sparse Bayesian learning and the relevance vector machine**. *Journal of Machine Learning Research* 2001, **1**:211-244.


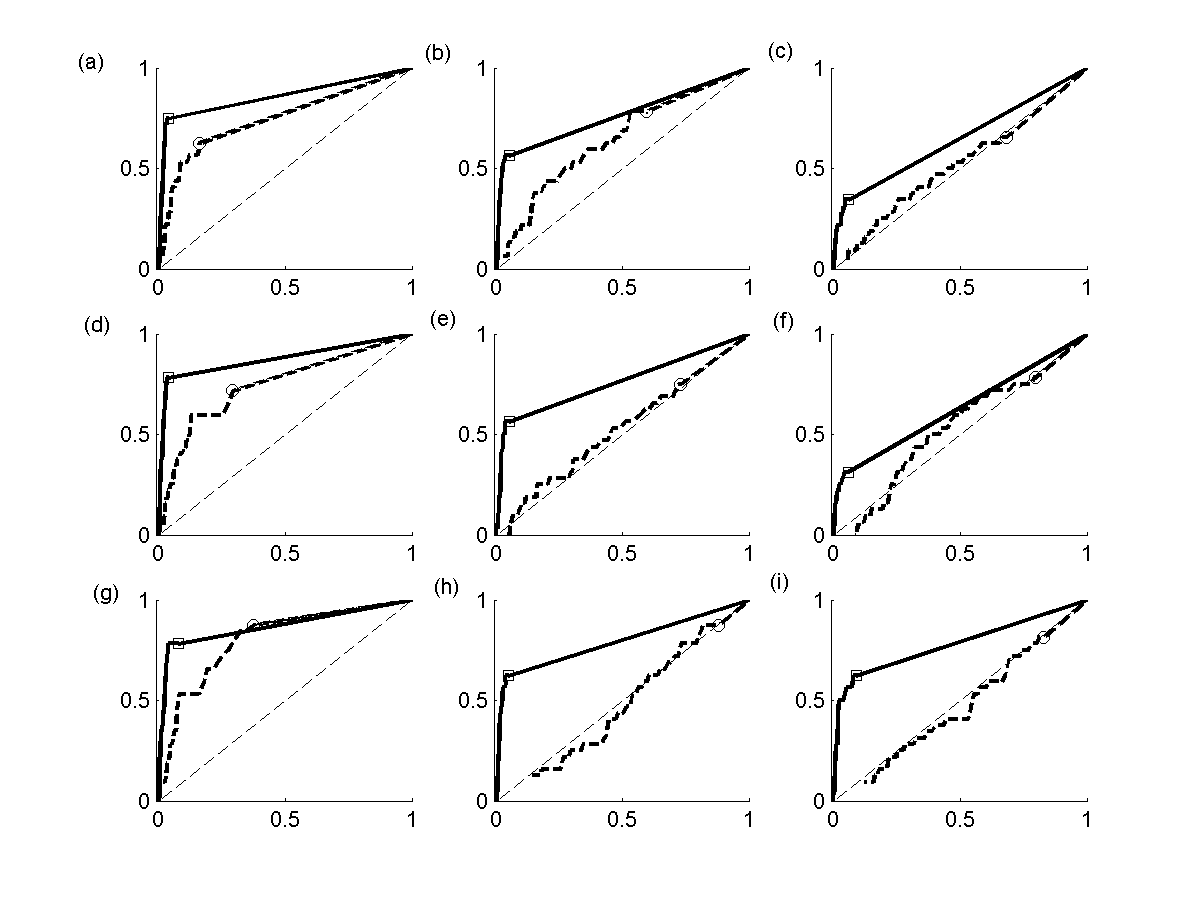


Figure S1. ROC comparison of BOLS (solid curve and square) and SBL (dotted curve and circle) output with K = 30. (a) R = 1 and ε = 0.01, (b) R = 1 and ε = 0.05, (c) R = 1 and ε = 0.1, (d) R = 2 and ε = 0.01, (e) R = 2 and ε = 0.05, (f) R = 2 and ε = 0.1, (g) R = 3 and ε = 0.01, (h) R = 3 and ε = 0.05, (i) R = 3 and ε = 0.1. For all Figures, the x-axis corresponds to the complementary specificity, the y-axis sensitivity.
